# Clinicopathological and prognostic significance of PKM2 protein expression in cirrhotic hepatocellular carcinoma and non-cirrhotic hepatocellular carcinoma

**DOI:** 10.1038/s41598-017-14813-y

**Published:** 2017-11-10

**Authors:** Yan Liu, Hao Wu, Ying Mei, Xiong Ding, Xiaoli Yang, Changping Li, Mingming Deng, Jianping Gong

**Affiliations:** 1 Department of Geriatric gastroenterology, the Fifth People’s Hospital of Chengdu, Sichuan, 611130 China; 2 Department of Hepatobiliary Surgery, the Second Affiliated Hospital of Chongqing Medical University, Chongqing, 400016 China; 3 Department of gastroenterology, the Affiliated Hospital of Southwest Medical University, Luzhou, 646000 China

## Abstract

Pyruvate kinase M2 (PKM2), a key protein in glucose and lipid metabolism, has been reported to be related to carcinogenesis in various malignancies. However, its roles in hepatocellular carcinoma with cirrhotic liver (CL) and hepatocellular carcinoma with non-cirrhoticliver (NCL) haves not been investigated. In our study western bloting, qRT-PCR and immunohistochemistry were performed to evaluate the clinical significance of PKM2 protein expression in CL and NCL. The results revealed that PKM2 protein expression was significantly higher in HCC tissues than in their adjacent non-tumour tissues. The high expression rates of PKM2 were more frequently noted in CL (45. 6%) than in NCL (31. 9%) tissues. High PKM2 expression in CL and NCL tissues was significantly associated with vascular invasion (P = 0.002 and P = 0.004, respectively) and intrahepatic metastasis (P < 0.001 and P = 0.019, respectively). Importantly, Kaplan–Meier survival analysis showed that the disease-specific survival (DSS) and recurrence-free survival (RFS) were lower in CL with high PKM2 expression than in NCL with high PKM2 expression (P = 0.003 and P = 0.003, respectively). Overall, high PKM2 expression was more frequently found in CL than in NCL, and PKM2 overexpression was associated with poor survival rates in patients with CL and NCL.

## Introduction

Hepatocellular carcinoma (HCC) is the fifth most common human cancer and represents the third most frequent cause of cancer deaths worldwide, with an estimated 745 000 deaths occurring annually^1–3^. Thus far, HCC is one of the few cancers with well-defined major risk factors, such as age and sex (male), hepatitis B and C virus, exposure to toxins (aflatoxin), chronic alcohol abuse and cirrhosis^4^. The majority of HCC patients often have liver cirrhosis, and the liver cirrhosis is caused by multiple internal and external factors, including alcohol consumption, hepatitis viruses, and fatty liver disease^5^. In the process of liver cirrhosis, liver cells experience chronic inflammation, fibrosis and scarring, and hepatocellular regeneration, resulting in accumulation of aberrant cells with genetic mutations causally associated with liver malignancy^6–8^. Chronic liver injury, typically cirrhosis, is the most important and common setting for the development of HCC. However, a portion of HCC cases have been known to occur in patients with a seemingly intact liver^9^. HCC patients with a non-cirrhotic liver (NCL)represent a relatively small proportion (10–30%) of HCC cases^10^. Non-cirrhotic HCC is characterized by a large tumour size, has a low risk of liver failure, is more receptive to hepatic and tumor resection and has a generally good prognosis compared to cirrhotic HCC^11,12^. However, HCC patients with a cirrhotic liver (CL)present with poorer pathological manifestation and worse prognosis, and the 5-year survival rate is only approximately 60% in hepatic resection or liver transplantation patients^5^. The prognosis of HCC patients with cirrhosis and non-cirrhosis is markedly different. Therefore, there might be different tumourigenic mechanisms between cirrhotic HCC and non-cirrhotic HCC, which highlights the importance of finding effective biomarkers to identify HCC with cirrhosis or non-cirrhosis and to provide personalized detection and therapeutic interventions to improve the survival rate and quality of life.

In the process of tumour growth and progression, changes in substance metabolism accompany uncontrolled cell proliferation^13^. Moreover, there is growing evidence that cancer is primarily a disease of energy metabolism, especially glucose metabolism^14,15^. Several recent studies have indicated that aerobic glycolysis (Warburg effect) plays a crucial role in the occurrence and development of tumours^16,17^. In glycolysis, glucose is enzymatically broken down to pyruvate by pyruvate kinase (PK), thus encouraging oxidative phosphorylation to aerobic glycolysis^18^. Pyruvate kinase in muscle cells (PKM) exists in two isoforms: PKM1 and PKM2. They are generated due to alternative splicing^19^. More specifically, pyruvate kinase M2 (PKM2), which converts phosphoenolpyruvate into pyruvate, is a key regulator of aerobic glycolysis because it is an enzyme that catalyses a crucial step^20^. In recent years, a review of the evidence indicates that high expression of PKM2 has been observed in numerous cancers, such as hilar cholangiocarcinoma, lung cancer, gastric cancer and colorectal cancers^21–24^. There is increasing evidence that PKM2 is involved in nutritional and metabolic neoplastic disease, but the associations among the protein expression level of PKM2, clinical significance, and the survival rate of HCC patients with cirrhosis or non-cirrhosis is unclear.

In the present study, we investigated the expression level of PKM2 in HCC with cirrhosis or non-cirrhosis and in paracancerous tissues. In addition, we further studied the relationship between PKM2 expression and clinicopathological factors and prognostic significance. We tried to find the influence of PKM2 expression the on the development of HCC with cirrhosis or non-cirrhosis and explored the possibility that PKM2 is a prognostic factors in HCC, and at the same time, we attempted to provide a reliable basis for scientific personalized treatment of HCC.

## Results

### PKM2 mRNA and protein expression in HCC

**Figure 1:**
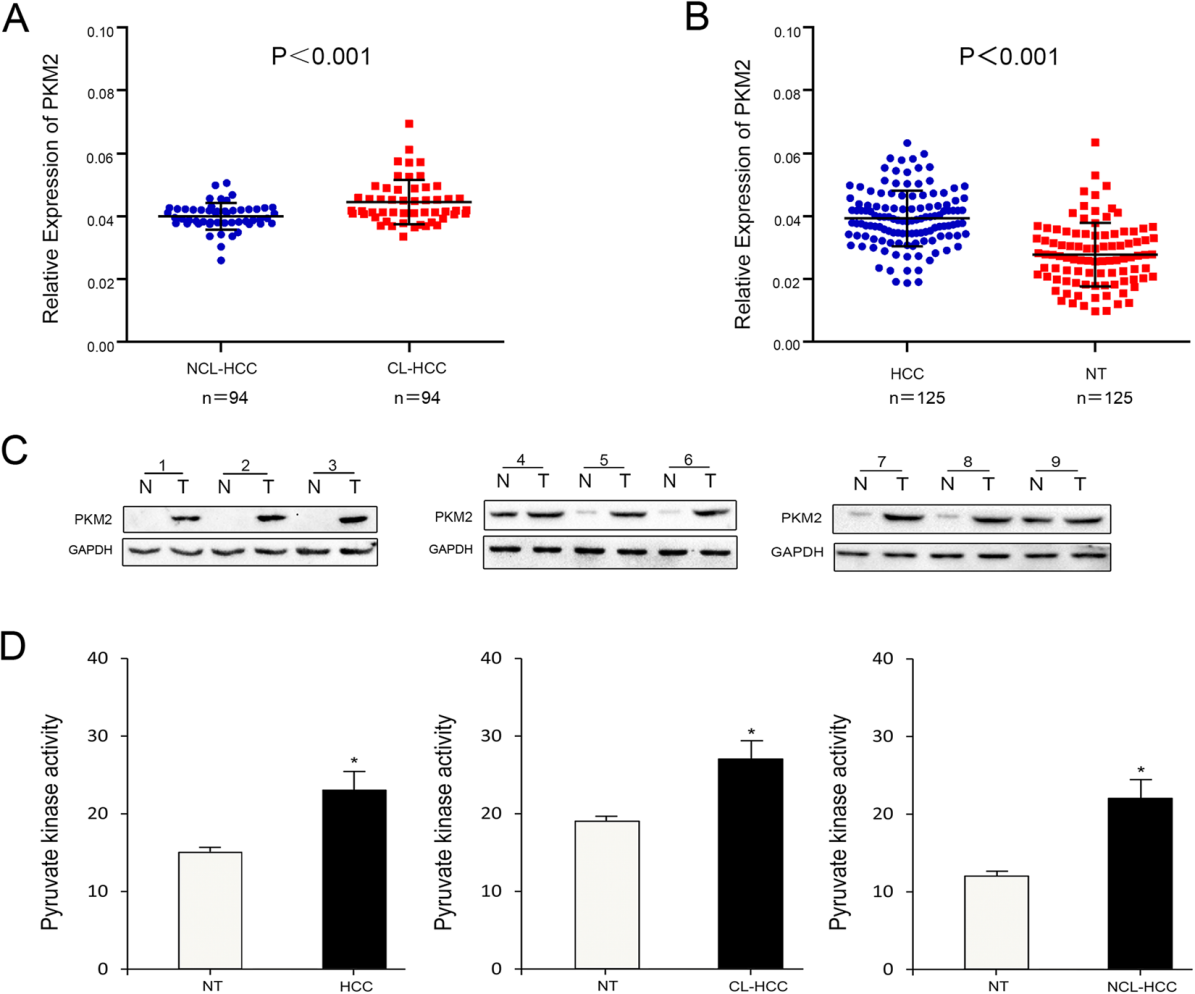
PKM2 mRNA, protein expression and enzyme activity in HCC. (**A**) The expression levels of PKM2 were measured with quantitative real-time PCR in 94 NCL-HCC and CL-HCC tissues. (**B**) The expression levels of PKM2 in the 125 HCC tumour and adjacent non-tumour liver tissues (HCC groups consist of randomly selected NCL-HCC and CL-HCC). (**C**) Protein levels of PKM2 in 9 representative HCC tissues (T) and adjacent non-tumour liver tissues (N) were analysed by western blotting. The uncropped blots details are provided in Supplementary Fig. S1. The relationship between PKM2 and GAPDH is provided in Supplementary Fig. S2. (**D**) Pyruvate kinase activity in HCC, CL-HCC and NCL-HCC, *P < 0.05.

**Figure 2:**
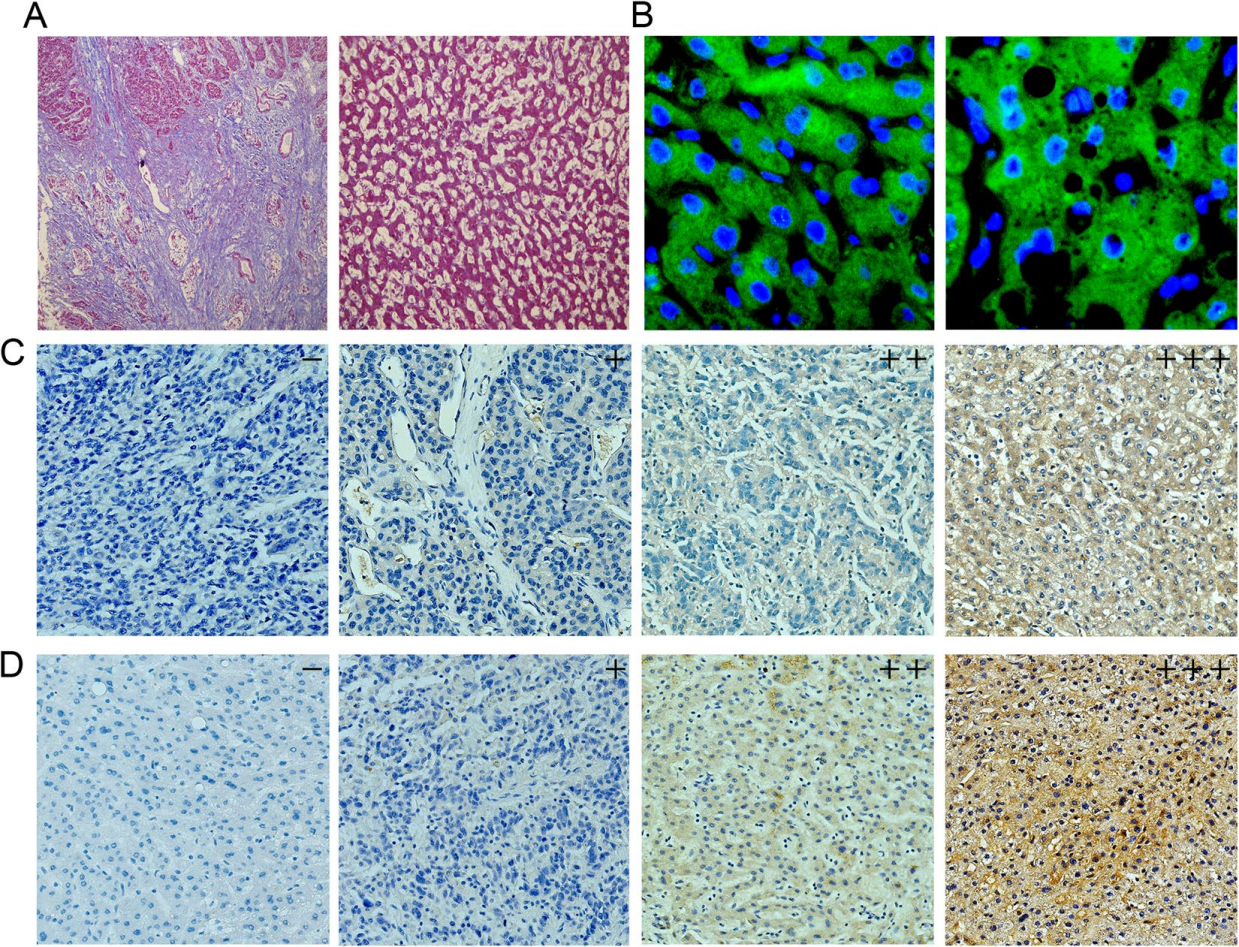
Masson’s trichrome staining and PKM2 expression in HCC tissue samples. (**A**) Masson’s staining in CL-HCC and NCL-HCC tissues (from left to right), ×200. (**B**) The sub-cellular localization of PKM2 in CL-HCC and NCL-HCC tissues (from left to right), ×200. (**C**) The expression levels of PKM2 in CL-HCC. (−) Negative expression; (+) Weak expression; (++) Moderate expression; (+++) High expression. Original magnification, ×200. (**D**) The expression levels of PKM2 in NCL-HCC. (−) Negative expression; (+) Weak expression; (++) Moderate expression; (+++) High expression. Original magnification, ×200.

We performed quantitative real-time PCR to examine tissue samples from all patients at the mRNA level. The PCR results showed that tumorous liver tissues exhibited increased PKM2 expression compared with the non-tumorous liver tissues (Fig. 1A,B). We also used western bloting to examine the expressions of PKM2 in each of 9 paired tumorous liver tissues and adjacent non-tumorous liver tissues in cirrhotic HCC and non-cirrhotic HCC. The results showed that tumorous liver tissues exhibited increased PKM2 expression compared with the adjacent non-tumorous liver tissues in cirrhotic HCC and non-cirrhotic HCC (Fig. 1C). We also found that the pyruvate kinase activity was higher in tumorous liver tissues than in non-tumorous liver tissues (P < 0.005) (Fig. 1D). Furthermore, we found a large number of fibre strands between HCC cells in cirrhotic HCC (Fig. 2A), and the expression of PKM2 was mainly concentrated in the cytoplasm (Fig. 2B). We confirmed PKM2 expression in cirrhotic HCC paraffin section and non-cirrhotic HCC paraffin sections. The immunohistochemistry results of indicated that high PKM2 expression was observed in cirrhotic HCC (45. 6%, 57/125) (Fig. 2C) and non-cirrhotic HCC (31. 9%, 30/94) (P < 0.005) (Fig. 2D). However, only high PKM2 expression was observed in the cirrhotic HCC adjacent non-tumour tissues (4%, 5/125) and non-cirrhotic HCC adjacent non-tumour tissues (3.1%, 3/94) (P > 0.005).

### The relationship between PKM2 and clinicopathological parameters of cirrhotic HCC and non-cirrhotic HCC patients

In our study, 125 cirrhotic HCC cases and 94 non-cirrhotic HCC cases were analysed. There were 32 female (25. 6%) and 93 male (74. 4%) cirrhotic HCC. 30 patients were <45 years old, and 95 patients were ≥45 years old. In the non-cirrhotic HCC group, 27 patients were female (28.7%), 67 patients were male (71.3%), and 27 patients were <45 years old, and 67 patients were ≥45 years old. Our results demonstrated that significant correlations between PKM2 expression in CL and NCL are both closely associated with vascular invasion (P = 0.002 and P = 0.004, respectively), and intrahepatic metastasis (P < 0.001 and P = 0.019, respectively). However, PKM2 status was not significantly associated with gender, age, HBV status, or multiplicity. The details of the basic finding are shown in Table 1.

**Table 1:** Patient clinicopathological characteristics and relationship with PKM2 expression.

Characteristics	N	Cirrhosis HCC		P value	N	Non-cirrhosis HCC		P value
		low	high			low	high	
**Gender**								
Female	32	18	14	0.808	27	19	8	0.763
Male	93	50	43		67	45	22	
**Age**								
<45	30	15	15	0.579	27	22	5	0.077
≥45	95	53	42		67	42	25	
**Tumor size (cm)**								
≤5	48	33	15	0.011	40	31	9	0.107
>5	77	35	42		54	33	21	
**AFP (ng/ml)**								
≤20	33	23	10	0.04	28	21	7	0.349
>20	92	45	47		66	43	23	
**HBsAg**								
Positive	110	61	49	0.521	57	39	18	0.622
Negative	15	7	8		37	25	12	
**Multiplicity**								
Single	82	49	33	0.097	59	40	19	0.938
Multiple (≥2)	43	19	24		35	24	11	
**Vascular invasion**								
Presence	67	28	39	0.002	52	29	23	0.004
Absence	58	40	18		42	35	7	
**Intrahepatic metastasis**								
Presence	59	21	38	<0.001	37	20	17	0.019
Absence	66	47	19		57	44	13	
**TNM stage**								
I/II	82	50	32	0.042	58	42	16	0.104
III/IV	43	18	25		36	22	14	
**Tumor Differentiation**								
poor	76	47	29	0.037	57	37	20	0.413
well	49	21	28		37	27	10	

P-values * were calculated using a chi-square (χ^2^) test. *P < 0.05.

### The relationship between high expression of PKM2 and poor disease-specific survival in cirrhotic HCC and non-cirrhotic HCC

**Figure 3:**
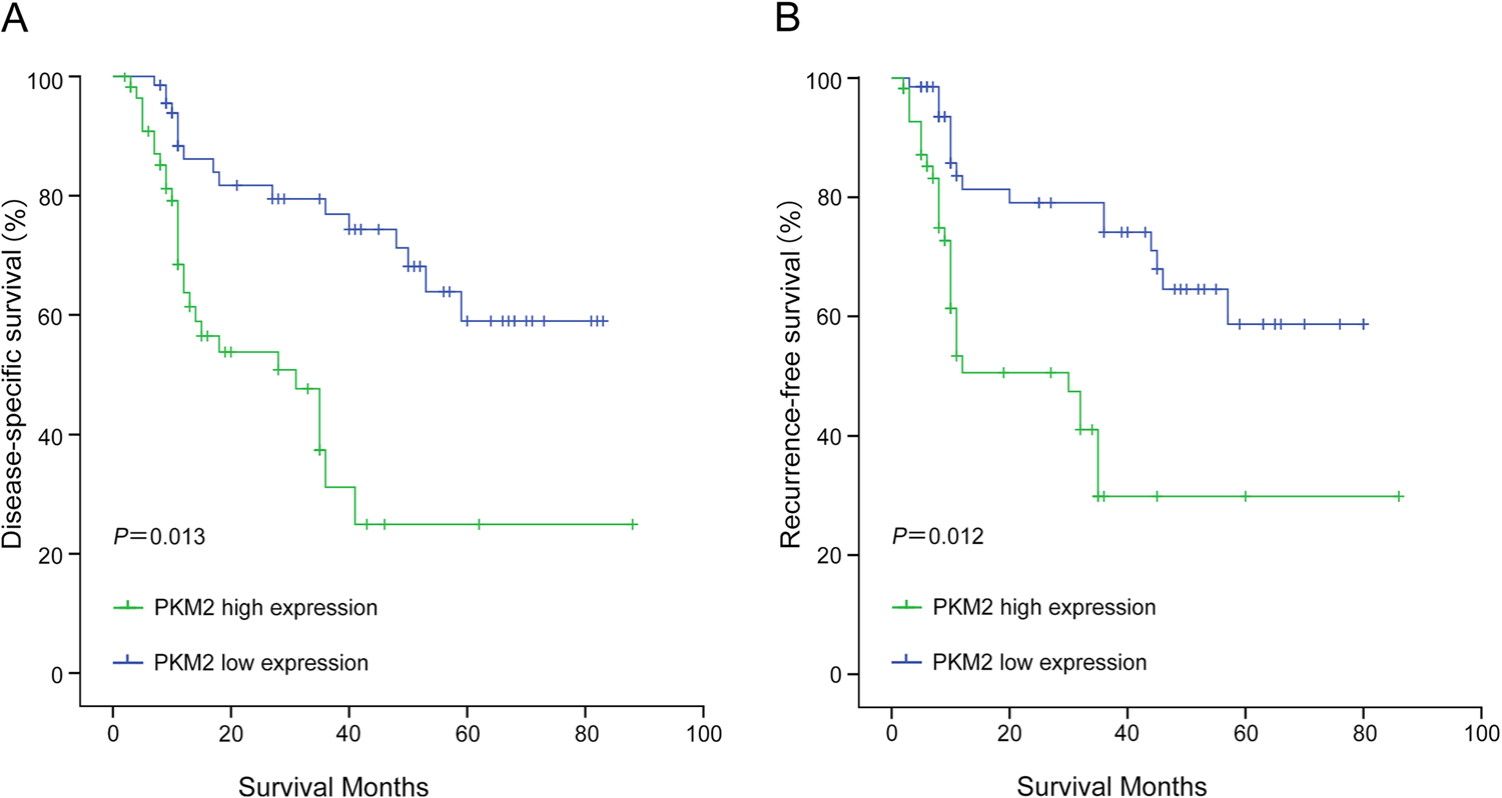
Survival analysis of PKM2 expression using the Kaplan-Meier method. (**A**) Kaplan–Meier survival curves of DSS in CL-HCC patients according to PKM2 expression. (**B**) Kaplan–Meier survival curves of RFS in CL-HCC patients according to PKM2 expression.

**Figure 4:**
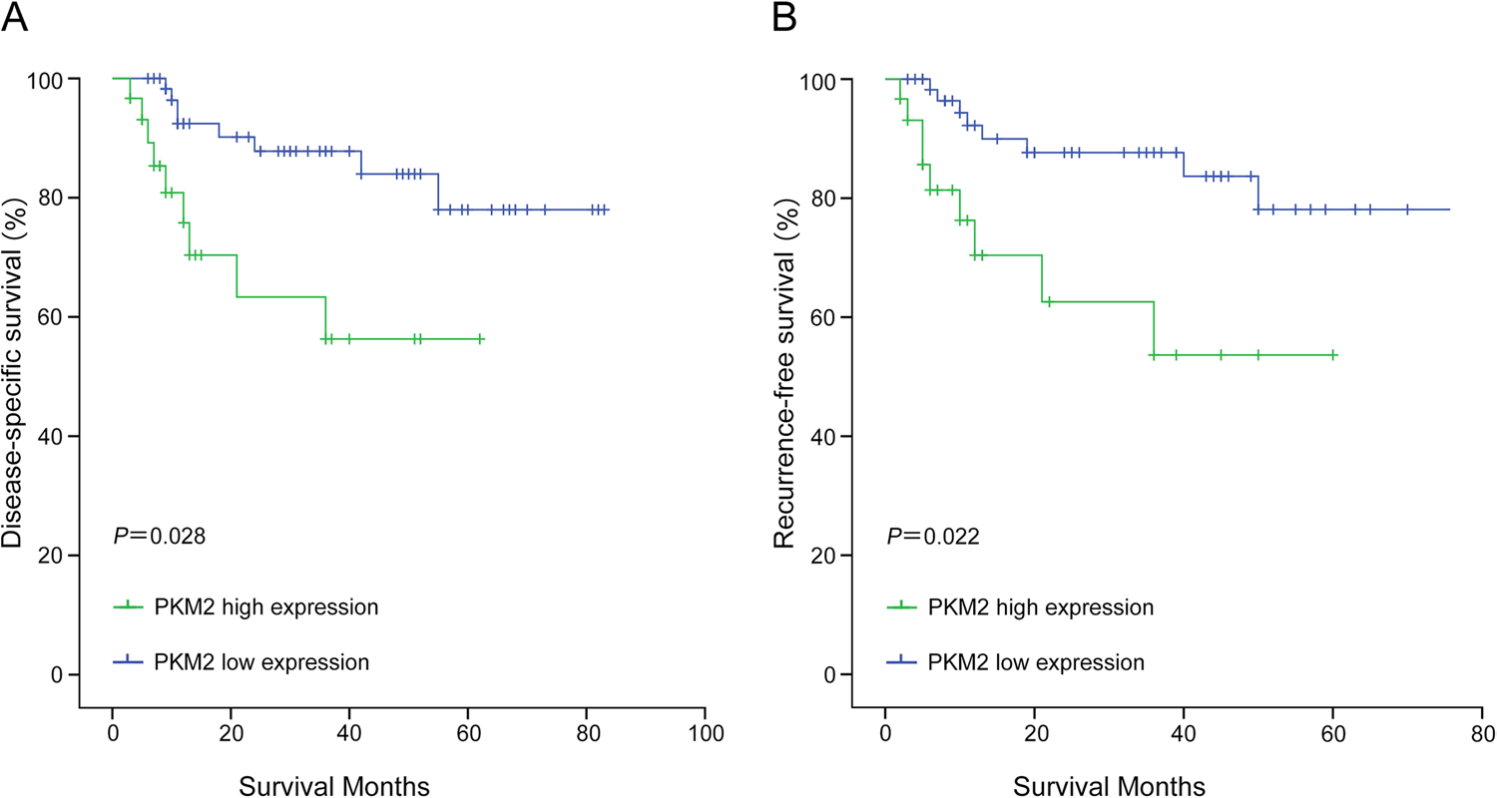
Survival analysis of PKM2 expression using the Kaplan-Meier method. (**A**) Kaplan–Meier survival curves of DSS in NCL-HCC patients according to PKM2 expression. (**B**) Kaplan–Meier survival curves of RFS in NCL-HCC patients according to PKM2 expression.

The cumulative survival curves were investigated using the Kaplan-Meier method, and differences in survival times were calculated according to the log rank test. The results show that the 1-year DSS rates in the low PKM2 expression group with cirrhotic HCC and non-cirrhotic HCC were 86. 1% and 86.6%, respectively, and the 3-year DSS rates were 74.8% and 78.3%, respectively. However, the 1-year DSS rates in the high PKM2 expression group with cirrhotic HCC and non-cirrhotic HCC were 73.8% and 75. 8%, respectively. The 3-year DSS rates were 41.0%and 56.3%, respectively. There were significant differences between the two groups in DSS (cirrhotic HCC: P = 0.013, non-cirrhotic HCC: P = 0.028) (Figs 3A and 4A).

**Figure 5:**
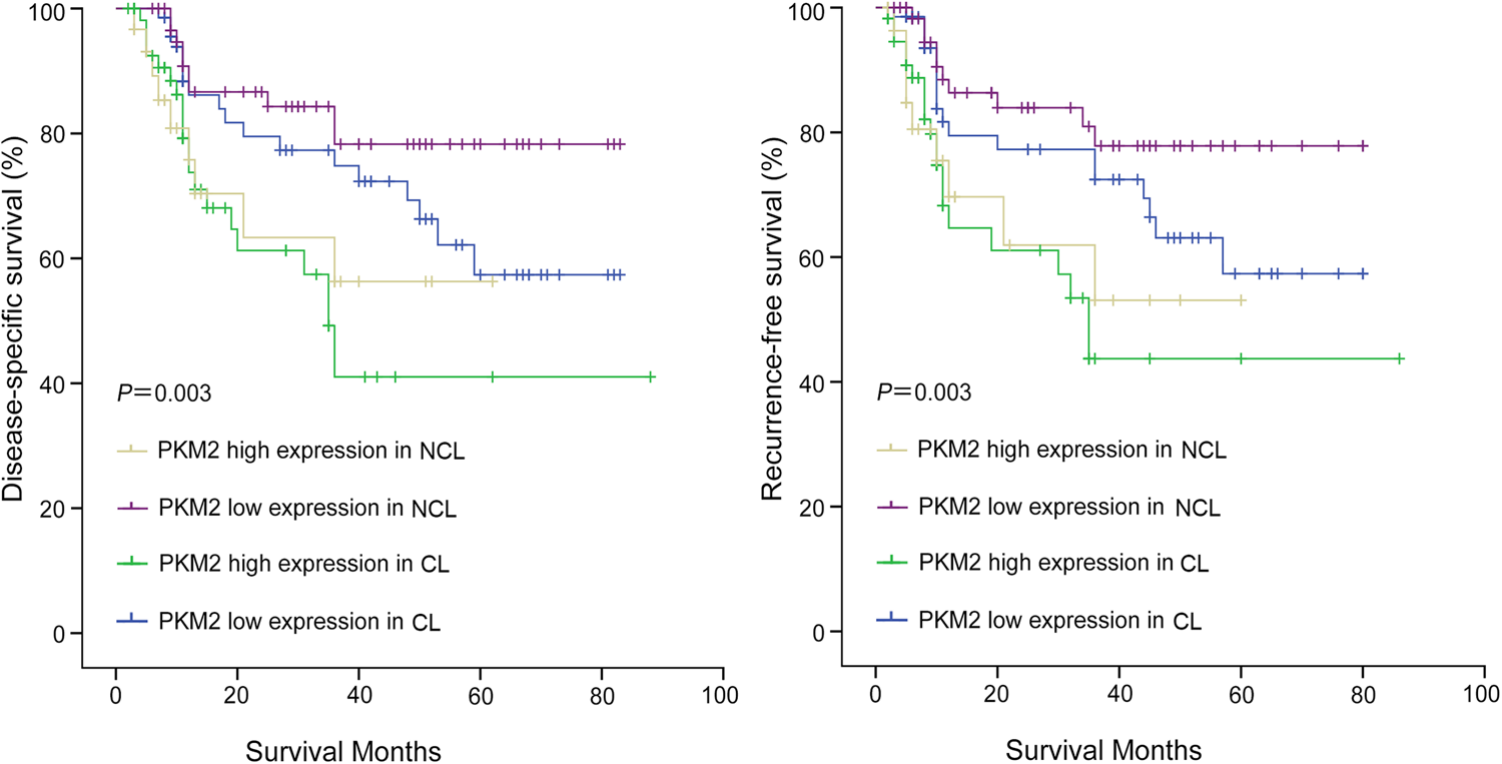
Survival analysis of PKM2 expression using the Kaplan-Meier method. (**A**) Kaplan–Meier survival curves of DSS between CL-HCC and NCL-HCC patients according to PKM2 expression. (**B**) Kaplan–Meier survival curves of RFS between CL-HCC and NCL-HCC patients according to PKM2 expression

When cirrhotic HCC and non-cirrhotic HCC patients were divided into two groups according to PKM2 expression, through comparative analysis, we found that the disease-specific survival rate was relatively lower in the high PKM2 expression group with cirrhotic HCC than in the high PKM2 expression group with non-cirrhotic HCC (P = 0.003) (Fig. 5A).

Univariate analysis revealed that alpha fetoprotein (HR = 1.526, P = 0.047), tumour size (HR = 1.475, P = 0.029), intrahepatic metastasis (HR = 2.191, P = 0.030), TNM stage (HR = 2.857, P = 0.034), vascular invasion(HR = 2.481, P = 0.029), tumour differentiation (HR = 1.753,P = 0.031) and PKM2 expression (HR = 2.240, P = 0.028) were significantly associated with DSS in cirrhotic HCC (Table 2). Tumour size (HR = 3.768, P = 0.036), intrahepatic metastasis (HR = 13.071, P = 0.027), TNM stage (HR = 1.255, P = 0.020), vascular invasion (HR = 1.078, P = 0.017) and PKM2 expression (HR = 5.126, P = 0.014) were adverse prognostic factors affecting DSS in non-cirrhotic HCC after resection (Table 3).

**Table 2:** Univariate analysis of different prognostic variables in DSS and RFS of CL-HCC patients using the by Cox proportional hazard model Abbreviations: 95% CI = 95% confidence interval; HR = hazard ratio; TNM stage = tumour node metastasis; P-value < 0.05.

Variables	DSS			RFS		
	HR	95%CI	P value	HR	95%CI	P value
**Sex**						
male/female	1.038	0.406–2.652	0.938	0.994	0.387–2.557	0.990
**Age(years)**						
≤50/>50	0.662	0.339–1.292	0.227	0.689	0.358–1.362	0.292
**AFP(ng/ml)**						
≤20/>20	1.526	0.279–2.990	0.047	0.528	0.279–1.001	0.050
**HBsAg**						
yes/no	0.761	0.252–2.297	0.628	0.737	0.243–2.234	0.059
**Tumor size(cm)**						
≤5/>5	1.475	0.543–1.927	0.029	3.415	1.214–3.805	0.009
**Multiplicity**						
Single/multiple	0.755	0.364–1.565	0.45	0.717	0.343–1.499	0.377
**Intrahepatic Metastasis**						
yes/no	2.191	0.943–3.854	0.03	1.212	0.748–1.942	0.041
**TNM stage**						
(I/II)/(III/VI)	2.857	1.084–7.531	0.034	3.380	1.269–9.003	0.015
**Vascular Invasion**						
yes/no	2.481	1.099–5.602	0.029	2.323	1.002–5.383	0.049
**Tumor Differentiation**						
Poor/well	1.753	0.910–2.356	0.031	1.203	1.215–1.781	0.019
**PKM2 expression**						
low/high	2.240	1.091–4.597	0.028	2.537	1.106–4.965	0.028

**Table 3:** Univariate analysis of different prognostic variables in DSS and RFS of NCL-HCC patients using the Cox proportional hazard model. Abbreviations: 95% CI = 95% confidence interval; HR = hazard ratio; TNM stage = tumour node metastasis; P-value < 0.05.

Variables	DSS			RFS		
	HR	95% CI	P value	HR	95% CI	P value
**Sex**						
male/female	0.494	0.101–2.431	0.386	0.528	0.109–2.548	0.426
**Age(years)**						
≤50/>50	0.983	0.306–3.163	0.977	1.145	0.356–3.678	0.821
**AFP(ng/ml)**						
≤20/>20	0.579	0.192–1.744	0.331	0.815	0.272–2.443	0.715
**HBsAg**						
yes/no	0.328	0.030–3.560	0.359	0.409	0.038–4.390	0.460
**Tumor size(cm)**						
≤5/>5	3.768	1.089–13.035	0.036	2.873	0.841–9.818	0.092
**Multiplicity**						
Single/multiple	0.462	0.061–3.501	0.455	0.440	0.059–3.275	0.423
**Intrahepatic Metastasis**						
yes/no	13.071	1.343–27.203	0.027	10.985	1.206–31.072	0.033
**TNM stage**						
(I/II)/(III/VI)	1.255	2.080–3.807	0.020	1.295	0.093–2.937	0.039
**Vascular Invasion**						
yes/no	1.078	1.010–2.635	0.017	1.116	0.015–1.866	0.036
**Tumor Differentiation**						
Poor/well	0.854	0.210–0.856	0.362	0.752	0.136–0.873	0.219
**PKM2 expression**						
low/high	5.126	1.383–9.003	0.014	4.100	4.178–14.268	0.027

In multivariate analysis, through analysis of the clinical significance of the research data, alpha fetoprotein and hepatitis B virus infection were considered to be closely related with HCC, Therefore, we established three models to carry on the multivariate analysis (Tables 4 and 5). For model 1, the research elements included alpha fetoprotein (AFP) and hepatitis B virus infection status, and the analysis results showed that AFP (HR = 1.412, P = 0.003) was an independent prognostic factor for DSS in patients with cirrhotic HCC; AFP and hepatitis B virus infection status were not prognostic factors for DSS in patients with non-cirrhotic HCC. For model 2, the research elements included AFP, hepatitis B virus infection status and PKM2 expression, and the analysis results showed that AFP (HR = 1.514, P = 0.028) and PKM2 expression (HR = 3.032, P = 0.001) were independent prognostic factors for DSS in patients with cirrhotic HCC; PKM2 expression (HR = 3.960, P = 0.007) was an independent prognostic factor for DSS in patients with non-cirrhotic HCC. Finally, for model 3, the research elements included AFP, hepatitis B virus infection status, tumour size, intrahepatic metastasis, TNM stage, vascular invasion, tumour differentiation and PKM2 expression, and the analysis results showed that tumour size (HR = 1.461, P = 0.019), TNM stage (HR = 3.011, P = 0.018), vascular invasion (HR = 1.895, P = 0.023), tumour differentiation (HR = 1.469, P = 0.043) and PKM2 expression (HR = 2.283, P = 0.034) were independent predictors of DSS in patients with cirrhotic HCC; tumour size (HR = 3.734, P = 0.027), intrahepatic metastasis (HR = 5.832, P = 0.011), TNM stage (HR = 1.260, P = 0.016), vascular invasion (HR = 1.108, P = 0.017), tumour differentiation (HR = 2.387, P = 0.033) and PKM2 expression (HR = 4.857, P = 0.007) were independent predictors of DSS in patients with non-cirrhotic HCC.

**Table 4:** Multivariate analysis of different prognostic variables in DSS and RFS of CL-HCC patients using the Cox proportional hazard model.

Variables	DSS			RFS		
	HR	95%CI	P value	HR	95%CI	P value
**Model 1**						
AFP(ng/ml)						
≤20/>20	1.412	0.230–1.737	0.003	1.423	0.237–0.756	0.004
HBsAg						
yes/no	0.913	0.326–2.554	0.628	0.958	0.343–2.678	0.925
**Model 2**						
AFP(ng/ml)						
≤20/>20	1.514	0.284–1.931	0.028	1.523	0.989–2.947	0.032
HBsAg						
yes/no	0.904	0.322–2.542	0.849	0.927	0.330–2.604	0.885
PKM2 expression						
low/high	3.032	1.599–5.749	0.001	3.020	1.602–5.695	0.001
**Model 3**						
AFP(ng/ml)						
≤20/>20	0.545	0.293–1.013	0.055	0.551	0.298–1.020	0.058
HBsAg						
yes/no	0.747	0.257–2.175	0.593	0.753	0.258–2.200	0.604
Tumor size (cm)						
≤5/>5	1.461	0.241–1.881	0.019	1.548	0.285–1.053	0.041
Intrahepatic Metastasis						
yes/no	0.254	0.060–1.073	0.062	1.236	0.0556–1.521	0.037
TNM stage						
(I/II)/(III/VI)	3.011	1.209–7.502	0.018	2.519	1.019–6.230	0.046
Vascular Invasion						
yes/no	1.895	0.889–3.993	0.023	2.051	0.988–4.256	0.034
Tumor Differentiation						
Poor/well	1.469	0.879–1.942	0.043	1.203	0.735–1.547	0.039
PKM2 expression						
low/high	2.283	1.067–4.888	0.034	2.181	1.054–4.513	0.036

Abbreviations: 95% CI = 95% confidence interval; HR = hazard ratio; TNM stage = tumour node metastasis; P-value < 0.05.

**Table 5:** Multivariate analysis of different prognostic variables in DSS and RFS of NCL-HCC patients using the Cox proportional hazard model.

Variables	DSS			RFS		
	HR	95%CI	P value	HR	95%CI	P value
**Model 1**						
AFP(ng/ml)						
≤20/>20	0.817	0.311–2.148	0.682	0.851	0.323–2.236	0.743
HBsAg						
yes/no	0.956	0.127–7.221	0.966	0.961	0.127–7.529	0.970
**Model 2**						
AFP(ng/ml)						
≤20/>20	0.875	0.331–2.313	0.788	0.935	0.354–2.471	0.892
HBsAg						
yes/no	0.634	0.080–4.994	0.665	0.638	0.081–5.025	0.669
PKM2 expression						
low/high	3.960	1.464–10.712	0.007	3.812	1.417–10.254	0.008
**Model 3**						
AFP(ng/ml)						
≤20/>20	0.627	0.217–1.815	0.390	0.862	0.298–2.494	0.784
HBsAg						
yes/no	0.247	0.025–2.422	0.230	0.306	0.032–2.932	0.304
Tumor size (cm)						
≤5/>5	3.734	1.160–12.020	0.027	2.904	0.899–4.381	0.045
Intrahepatic Metastasis						
yes/no	5.832	1.980–17.095	0.011	5.354	1.810–10.261	0.012
TNM stage						
(I/II)/(III/VI)	1.260	0.487–1.776	0.016	2.276	1.093–3.818	0.020
Vascular Invasion						
yes/no	1.108	0.217–1.676	0.017	1.312	1.021–3.819	0.030
Tumor Differentiation						
Poor/well	2.387	1.302–2.406	0.033	1.891	1.520–3.845	0.028
PKM2 expression						
low/high	4.857	1.534–15.375	0.007	3.996	1.314–12.154	0.015

Abbreviations: 95% CI = 95% confidence interval; HR = hazard ratio; TNM stage = tumour node metastasis; P-value < 0.05.

### The relationship between high expression of PKM2 and poor recurrence-free survival in cirrhotic HCC and non-cirrhotic HCC

The 1-year RFS rates in the low PKM2 expression group with cirrhotic HCC and non-cirrhotic HCC were 79. 5% and 86.3%, respectively, and the 3-year RFS rates were 72.4% and 77.8%, respectively. However, the 1-year RFS rates in the high PKM2 expression group with cirrhotic HCC and non-cirrhotic HCC were 64.7% and 69.7%, respectively. The 3-year RFS rates were 42.8%and 53.1%, respectively. There was a significant difference between the two groups in RFS (cirrhotic HCC: P = 0.012, non-cirrhotic HCC: P = 0.022) (Figs 3B and 4B).

When cirrhotic HCC and non-cirrhotic HCC were divided into two groups according to PKM2 expression, through comparative analysis, we found that the recurrence-free survival rate was relatively lower in the high PKM2 expression group with cirrhotic HCC than in the high PKM2 expression group with non-cirrhotic HCC (P=0.003) (Fig. 5B).

Univariate analysis revealed that tumour size (HR = 3.415, P = 0.009), intrahepatic metastasis (HR = 1.212, P = 0.041), TNM stage (HR = 3.380,P = 0.015), vascular invasion (HR = 2.323, P = 0.049), tumour differentiation (HR = 1.203, P = 0.019) and PKM2 expression (HR = 2.537, P = 0.028) were significantly associated with RFS in cirrhotic HCC (Table 2). Intrahepatic metastasis (HR = 10.985, P = 0.033), TNM stage (HR = 1.295, P = 0.039), vascular invasion (HR = 1.116, P = 0.036) and PKM2 expression (HR = 4.100, P = 0.027) were adverse prognostic factors affecting RFS in non-cirrhotic HCC after resection (Table 3).

In multivariate analyses, we still used the three different Cox models to analyse the significance of PKM2 for RFS in cirrhotic HCC and non-cirrhotic HCC (Tables 4 and 5). For model 1, the research elements included alpha fetoprotein (AFP) and hepatitis B virus infection status, and the analysis results showed that AFP (HR = 1.423, P = 0.004) was an independent prognostic factor for RFS in patients with cirrhotic HCC; AFP and hepatitis B virus infection status were not prognostic factors for RFS in patients with non-cirrhotic HCC. For model 2, the research elements included AFP, hepatitis B virus infection status and PKM2 expression, and the analysis results showed that AFP (HR = 1.523, P = 0.032) and PKM2 expression (HR = 3.020, P = 0.001) were independent prognostic factors for RFS in patients with cirrhotic HCC; PKM2 expression (HR = 3.812, P = 0.008) was an independent prognostic factor for RFS in patients with non-cirrhotic HCC. Finally, for model 3, the research elements included AFP, hepatitis B virus infection status, tumour size, intrahepatic metastasis, TNM stage, vascular invasion, tumour differentiation and PKM2 expression, and the analysis results showed that tumour size (HR = 1.548, P = 0.041), intrahepatic metastasis (HR = 1.236, P = 0.037), TNM stage (HR = 2.519, P = 0.046), vascular invasion (HR = 2.051, P = 0.034), tumour differentiation (HR = 1.203, P = 0.039) and PKM2 expression (HR = 2.181, P = 0.036) were independent predictors of RFS in patients with cirrhotic HCC; tumour size (HR = 2.904, P = 0.045), intrahepatic metastasis(HR = 5.354, P = 0.012), TNM stage (HR = 2.276, P = 0.020), vascular invasion (HR = 1.312, P = 0.030), tumour differentiation (HR = 1.891, P = 0.028) and PKM2 expression (HR = 3.996, = 0.015) were independent predictors of RFS in patients with non-cirrhotic HCC.

## Discussion

Many studies have demonstrated that abnormal glucose metabolism is an important feature of tumour^25,26^. Regardless of the presence of oxygen, the tumour cells mainly produce energy through glycolysis. The PKM2 gene, located on chromosome 15q22, is a key rate-limiting enzyme in glycolysis and was found to be highly expressed in proliferating cells, especially in tumour cells, and PKM2 upregulation is believed to play a vital role in the Warburg effect and the occurrence a and development of tumors^27^. However, accurate prediction of the overall survival rates of patients with cirrhosis HCC or non-cirrhosis HCC is still unsatisfactory. Therefore, according to previous studies, we tried to explain the clinicopathological effects and prognostic significance of PKM2 protein expression in cirrhotic HCC and non-cirrhotic HCC. In the present study, we found that PKM2 was more positively expressed in HCC (57.1%) than in adjacent tissues (5.1%) and more frequently expressed in cirrhotic HCC (45.6%) than in non-cirrhosis HCC (31.9%).Meanwhile, PKM2- high expression patients have a poor prognosis. Our results demonstrate that PKM2 is a potentially valuable biomarker to predict the recurrence and survival interval after surgical resection.

In recent years, several studies have indicated that PKM2 is a crucial oncogene in a number of human primary tumours, including lung cancer, cervical cancer, glioblastoma and colorectal cancer^28–31^. In a study based on 16 months median follow-up of 88 hilar cholangiocarcinoma patients, HK1 and PKM2 levels were found to be higher in human hilar cholangiocarcinoma tumours than in normal tissue samples, and this expression was correlated with lymph node metastasis, tumour differentiation, TNM stage and poor prognosis^32^. In a previous study that included 266 cases of pancreatic cancer, the results showed that 73% of tumours expressed PKM2 in all cell compartments^33^. Recent studies have shown that over-expression of PKM2 occours in human HCC^34,35^. Based on a tissue microarray analysis that included samples from 668 HCC patients with complete clinical records and matched normal samples, Chen al. suggested that PKM2 was an independent prognostic indicator of recurrence-free or overall survival of HCC patients and PKM2 expression was clinicopathologically associated with vascular invasion, tumour number and TNM stage^36^. In our study, we found that advanced PKM2 expression was a poor independent prognostic indicator and positively related to unfavourable disease-specific survival and recurrence-free survival after surgical dissection. Importantly, we demonstrated that cirrhosis-associated HCC exhibits higher positive PKM2 expression rates than non-cirrhosis-associated HCC. Moreover, the disease-specific survival and recurrence-free survival were lower in cirrhosis HCC than non-cirrhosis HCC with high PKM2 expression. The results of these studies indicate that PKM2 plays an important role in early diagnosis of cancer, evaluation of the effect of tumour and the prognosis of patients.

PKM2 exists in catalytically distinct tetrameric and dimeric states^37^. Tetrameric PKM2 is mainly in the cytoplasm and participates in synthesis and catabolism of ATP. Dimeric PKM2 is found in cancer cells and is involved in synthesis of nucleic acids and amino acids. PKM2 goes into the nucleus and interacts directly with the HIF-1α subunit and promotes transactivation of HIF-1 target genes by enhancing HIF-1 binding and recruitment of p300, a transcriptional coactivator, which regulates HIF-1 activity. Transcription of the PKM2 gene is also activated by HIF, creating a positive feedback loop that promotes HIF activation and changes glucose metabolism in cancer cells^38,39^. Dimeric PKM2 can directly phosphorylate signal transducer and activator of transcription 3 (STAT3), which activates inflammatory cytokines, including interleukin-6^40^. PKM2 phosphorylates STAT3 at tyrosine-705 using phosphoenolpyruvate as a phosphate donor, which activates the transcription of mitogen-activated protein kinase 5. The activation of STAT3 in malignant tumour cells may be one of the important molecular markers for tumour progression^41^. Studies have shown that translocation of PKM2 may activate EGFR^42^. In PKM2, lysine-433 binds to c-Src-phosphorylated tyrosine-333 on β-catenin, and this results in removal of histone deacetylase 3 (HDAC3) from the promoter, histone H3 acetylation and CCND1 expression^43^. The transcription of PKM2- dependent β-catenin is required for EGFR promotion of tumour cell proliferation and tumour development^44^. Recently, a study reported that when the membrane receptor tyrosine kinase (RTKs) -PI3K/AKT-mTOR signalling pathway is over activated, it results in up regulation of PKM2 expression, which leads to an increase in the cellular Warburg effect. The Warburg effect is caused by mTOR using HIF1-mediated PKM gene transcription and c-Myc mediated selective shear to increase the expression of PKM2, which promotes the occurrence and development of tumours^45^.

A significant feature of malignant tumours cells is that they can switch from oxidative phosphorylation to aerobic glycolysis, known as the Warburg effect, which leads to tumourigenesis and cancer cell proliferation^46^. Meanwhile, mutation of anti-oncogenes is linked to regulation of proliferation, the cell cycle, and apoptosis and to genetic stability^47^. PKM2, through dephosphorylation of Cdc25A, promotes PKM2-dependent β-catenin transactivation and c-Myc-mediated upregulation of the expression of the glycolytic genes GLUT1, PKM2 and LDHA. These proteins promote the Warburg effect, cell proliferation and tumourigenesis^48^.When mitogenic and oncogenic proteins are stimulated by K433 acetylation, FBP binding is inhibited, preventing allosteric activation, and this activates PKM2 protein kinase activity and nuclear localization^49^. Simultaneously, PKM2 is also regulated by K305 acetylation. Acetylation under high-glucose stimulation targets PKM2 for degradation through chaperone-mediated autophagy and promotes tumour growth^50^. It was also reported that PKM2 induces EGFR phosphorylation and activates downstream EGFR signalling in triple-negative breast cancer cells^51^. We found that high PKM2 expression was correlated with vascular invasion and intrahepatic metastasis in our study. More specifically, cirrhosis HCC exhibited higher PKM2 expression and lower survival rates than non-cirrhosis HCC. This demonstrates that high PKM2 expression in tumour tissue can promote tumour cell proliferation and the malignant degree of cancer cells.

In conclusion, in our retrospective study, we are the first to find that PKM2 expression is higher in cirrhosis HCC than in non-cirrhosis HCC. The clinical data analysis indicated that increased PKM2 expression in HCC was correlated with poor prognostic indicators, such as vascular invasion and intrahepatic metastasis. Moreover, cirrhosis HCC, which is more malignant and invasive, has high PKM2 levels were strongly correlated with AFP, multiplicity, TNM stage and tumour differentiation. It is clear that positive PKM2 expression indicates aggressiveness and poor prognosis of HCC. At the same time, the survival analysis results showed that positive PKM2 expression was an independent poor prognostic factor for RFS and DSS. In addition, high PKM2 expression in cirrhosis HCC indicates poorer survival than that in non-cirrhosis HCC. These findings suggest that PKM2 plays an important role in the occurrence and development of tumour and is also an important cause of the tumour malignancy. To the best of our knowledge, this is the first study of PKM2 expression in cirrhosis HCC and non-cirrhosis HCC. Of course, our research is limited, and further studies should investigate the specific mechanism of PKM2 signalling in cirrhosis HCC and non-cirrhosis HCC progression, such as the ways of PKM2 converted to distinct tetrameric and dimeric states, the specific mechanism of dimeric PKM2 entry into the nucleus and the relation of PKM2 and oncogenic signaling pathways. Provide a scientific basis for molecular diagnosis and targeted therapy of cirrhosis HCC and non-cirrhosis HCC.

## Materials and Methods

### Ethics Statement

The research protocol was reviewed and approved by the Research Ethics Committee of the Second Affiliated Hospital of Chongqing Medical University. All experiments were conducted in accordance with approved guidelines of the Second Affiliated Hospital of Chongqing Medical University.

### Patients and tissue samples

In our study, all patient tissue samples were collected from May 2004 to May 2010 in the Second Affiliated Hospital of Chongqing Medical University. A total of 219 patients receiveing elective surgery for hepatocellular carcinoma were analysed. These samples included 125 cirrhotic HCC cases and 94 non-cirrhotic HCC cases. All surgical resection specimens were immediately processed after surgical removal, were fixed with 4% paraformaldehyde (pH 7.4) and embedded in paraffin for no longer than 24 h. The histopathological diagnosis of cirrhotic HCC and non-cirrhotic HCC was performed by two experienced independent pathologists in the Department of Pathology Archives of the Second Hospital Affiliated to Chongqing Medical University using haematoxylin and eosin (HE) staining. The diagnosis of cirrhotic HCC and non-cirrhotic HCC was made according to the World Health Organization (WHO) criteria for the study of liver disease.The complete clinical and prognostic data of the study were recorded accurately. The content of the analysis included: age at diagnosis, sex, tumour size, distant metastases, cirrhosis, hepatitis B virus infection, and serum AFP levels (ng/ml), which were obtained from patient medical records. Up to the deadline of May 2016, all respondents with cirrhotic HCC and non-cirrhotic HCC underwent follow-up. The tumour stages were defined according to the seventh edition of the American Joint Committee on Cancer Staging manual. All donors provided written informed consent to donate their samples. The study was performed according to the guidelines of the Declaration of Helsinki and the guidelines of the Ethics Review Committee of the Second Affiliated Hospital of Chongqing Medical University, and each patient signed an informed consent form.

### Western blot analysis

The protein in each sample was extracted through tissue homogenation. Similar protein concentrations were loaded into the gel, separated by SDS-PAGE gels electrophoresis (8–12% SDS–PAGE gels), and transferred to a nitrocellulose membrane using the wet transfer method (Bio-Rad, Hercules, CA, USA). The membranes were blocked with TBST (0.05% Tween-20 in TBS) containing 5% skim milk and then incubated overnight with PKM2 (ab150377, Abcam Inc., Cambridge, CA, USA) (1:1000) and GAPDH(ab8245, Abcam Inc., Cambridge, CA, USA) (1:5000) antibodies at 4 °C overnight. Next, the membranes were washed three times in TBST, and then incubated with horseradish peroxidase-conjugated goat anti-rabbit or anti-mouse secondary antibodies(Bio-Rad, Hercules, CA, USA) (1:5000) for 60 minutes at room temperature. Western Blotting Lightning Reagent (203-15291; PerkinElmer) was used to detect the results.

### Quantitative real-time PCR

Total RNA was extracted from tissues with Trizol reagent (Takara, Shiga, Japan) according to the manufacturer’s instructions. Primers targeting PKM2 and β-actin (internal control) were synthesized (Shengong Bio, Shanghai, China). Reverse-transcription PCR (RT-PCR) was performed according to instruction provided in a Prime-Script RT Reagent Kit (TaKaRa, Dalian, China). The primer sequences used to amplify PKM2 were 5′-AAGGGTGTGAACCTTCCTGG-3′ and 5′-GCTCGACCCCAAACTTCAGA-3′; β-actin was used as an internal control for normalization.

### Immunohistochemistry and evaluation

Briefly, the paraffin-embedded sections (4 μm) were deparaffinized in xylene, and rehydrated in a 100%, 95%, and 85% gradient ethanol series, Antigen retrieval was performed in 10mm citric buffer (pH 6.0) at 100 °C for 15 min. After slides were washed with PBS (pH 7.2), endogenous peroxidase activity was blocked with 3% H_2_O_2_ for 15 min. Then, nonspecific protein binding was blocked with goat serum for 15 min at ambient temperature, and the slides were incubated in a moist chamber with antihuman PKM2 rabbit monoclonal antibody at a 1:1000 dilution (rabbit monoclonal antibody, ab38237, 1:1000, Abcam Inc., Cambridge, CA, USA) at 4 °C overnight. Next, after being washed three times in PBS (pH 7.2), slides were incubated with biotinylated secondary antibody at 37 °C for 15 min and visualized with avidin horseradish enzyme at 37 °C for 20 min. The peroxidase reaction was performed using DAB (3,3- diaminobenzidine) for 20 seconds and rinsing in water for 10 min, and then, samples were counterstained with 1% Mayer’s haematoxylin solution, dehydrated in a gradient ethanol series and sealed with neutral gum. To quantify liver fibrosis, the blue pixel content of the images was photographed using the same microscope and magnification times. A semi-quantitative assessment method of scoring was used to judge the expression of PKM2. The evaluation system included staining intensity and the percentage of positive cells, where staining intensity ranged from 0–3 (0, negative; 1, weak; 2, moderate; 3, strong) and the percentage of positive cells ranged from 0–4 (0, negative or <5%1, 6–25%; 2, 26–50%; 3, 51–75%; and 4, 76–100%). The positive cell percentage scores and the intensity scores were summed to determine the expression of PKM2. Final staining scores <4 were identified as low PKM2 expression, while scores ≥4 were identified as high PKM2 expression.

### Measurement of pyruvate kinase activity

Pyruvate kinase activity was measured by Pyruvate Kinase Assay Kit (Abcam, Cambridge, MA, USA) and according to the manufacturer’s instructions.

### Co-immunoprecipitation (co-IP) assays

The protein in each sample was extracted through tissue homogenation. The co- immunoprecipitated using the Co-IP Kit (Pierce) according to the manufacturer’s instructions. The immunoprecipitates were washed four times with lysis buffer and stored at −80 °C until needed or were boiled with Laemmli sample buffer, followed by SDS–PAGE and western blotting.

### Statistical Analysis

All the experiment data were repeated at least three times. The research data are presented as the mean ± standard deviation for normally distributed continuous variables, and count data are presented as the frequency or percentage for categorical variables. The difference in mRNA expression of PKM2 was determined by wilcoxon matched-pairs signed rank test. Fisher’s exact test or chi square test was used to evaluate the relationships between PKM2 expression and clinicopathological parameters in cirrhotic HCC and non-cirrhotic HCC. The differences in survival between cirrhotic HCC and non-cirrhotic HCC were compared using the Kaplan-Meier method (log-rank test). Univariate and multivariate Cox regression analyses were performed to study the effects of PKM2 expression on different variables in cirrhotic HCC and non-cirrhotic HCC.*P* < 0.05 was the threshold for statistical significance.

### Ethical approval

All procedures performed in studies involving human participants were in accordance with the ethical standards of the ethical committee of the Second Affiliated Hospital of Chongqing Medical University and with the 1964 Helsinki Declaration and its later amendments or comparable.

### Ethical standards

Informed consent was obtained from all individual participants included in the study.

## Electronic supplementary material

Protein levels of PKM2 in 9 representative HCC tissues

The relationship between PKM2 and GAPDH
