# Supplementary material for: Clinicopathological and prognostic significance of PKM2 protein expression in cirrhotic hepatocellular carcinoma and non-cirrhotic hepatocellular carcinoma
Source: Sci Rep. 2017 Nov 10;7:15294. doi: 10.1038/s41598-017-14813-y (PMC5681582; doi:10.1038/s41598-017-14813-y)
Supplement: Supplementary file 2 — The relationship between PKM2 and GAPDH [file 41598_2017_14813_MOESM2_ESM.pdf]

Clinicopathological and prognostic significance of PKM2 protein expression in cirrhotic hepatocellular carcinoma and non-cirrhotic hepatocellular carcinoma

Yan Liu[1, *], **Hao Wu**[2, *], **Ying Mei**[2], **Xiong Ding**[2], **Xiaoli Yang**[2], **Changping Li**[3], **Mingming Deng**[3], **Jianping Gong**[2]

[1]Department of Geriatric gastroenterology, the Fifth People's Hospital of Chengdu, Sichuan, 611130, China.[2]Department of Hepatobiliary Surgery, the Second Affiliated Hospital of Chongqing Medical University, Chongqing 400016, China. [3]Department of gastroenterology, the Affiliated Hospital of Southwest Medical University, Luzhou 646000, China.*These authors contributed equally to this work. Correspondence and requests for materials should be addressed to C.L. (email:506854209@qq.com) or M. D. (email:793070544@qq.com) or J.G. (email: gongjianping11@126.com)

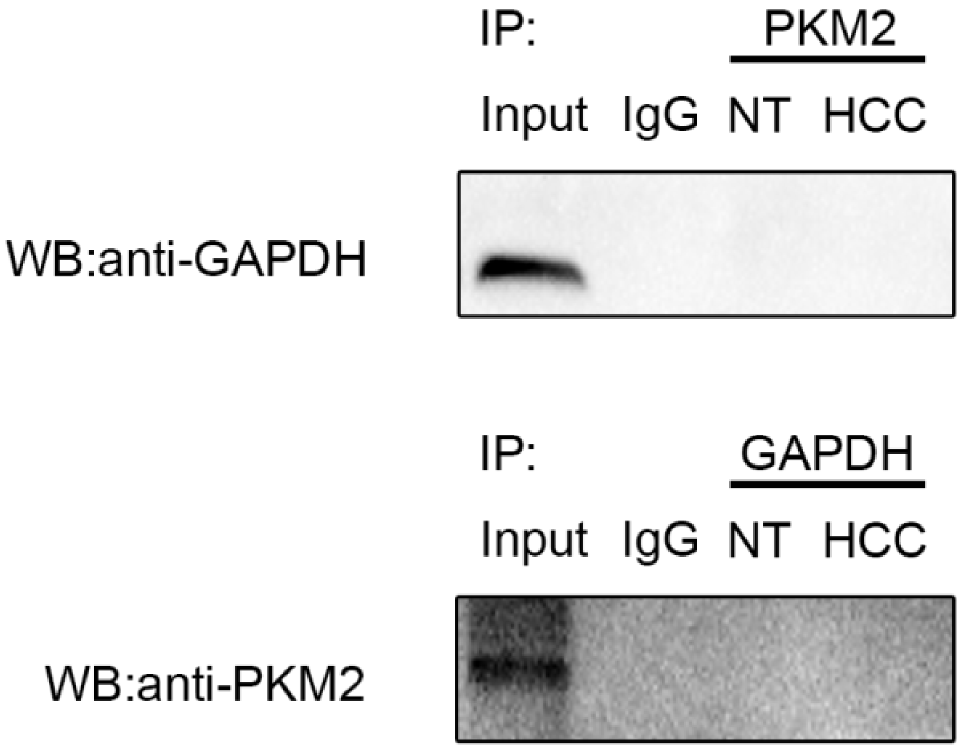
IP:
PKM2
Input
IgG
NT
HCC
WB:anti-GAPDH
IP:
GAPDH
Input
IgG
NT
HCC
WB:anti-PKM2
